# Accelerometer-based physical activity in a large observational cohort - study protocol and design of the activity and function of the elderly in Ulm (ActiFE Ulm) study

**DOI:** 10.1186/1471-2318-10-50

**Published:** 2010-07-27

**Authors:** Michael D Denkinger, Sebastian Franke, Kilian Rapp, Gudrun Weinmayr, Enric Duran-Tauleria, Thorsten Nikolaus, Richard Peter

**Affiliations:** 1 Agaplesion Bethesda Clinic, Ulm, Germany; 2 Institute of Epidemiology, Ulm University, Ulm, Germany; 3 Epidemiology and Public Health Unit, Institut de Prestacions d'Assitència Mèdical Personal Municipal (PAMEM), Barcelona, Spain; 4 IMIM - Hospital el Mar, Barcelona, Spain

## Abstract

**Background:**

A large number of studies have demonstrated a positive effect of increased physical activity (PA) on various health outcomes. In all large geriatric studies, however, PA has only been assessed by interview-based instruments which are all subject to substantial bias. This may represent one reason why associations of PA with geriatric syndromes such as falls show controversial results. The general aim of the Active-Ulm study was to determine the association of accelerometer-based physical activity with different health-related parameters, and to study the influence of this standardized objective measure of physical activity on health- and disability-related parameters in a longitudinal setting.

**Methods:**

We have set up an observational cohort study in 1500 community dwelling older persons (65 to 90 years) stratified by age and sex. Addresses have been obtained from the local residents registration offices. The study is carried out jointly with the IMCA - Respiratory Health Survey in the Elderly implemented in the context of the European project IMCA II. The study has a cross-sectional part (1) which focuses on PA and disability and two longitudinal parts (2) and (3). The primary information for part (2) is a prospective 1 year falls calendar including assessment of medication change. Part (3) will be performed about 36 months following baseline. Primary variables of interest include disability, PA, falls and cognitive function. Baseline recruitment has started in March 2009 and will be finished in April 2010.

All participants are visited three times within one week, either at home or in the study center. Assessments included interviews on quality of life, diagnosed diseases, common risk factors as well as novel cognitive tests and established tests of physical functioning. PA is measured using an accelerometer-based sensor device, carried continuously over a one week period and accompanied by a prospective activity diary.

**Discussion:**

The assessment of PA using a high standard accelerometer-based device is feasible in a large population-based study. The results obtained from cross-sectional and longitudinal analyses will shed light on important associations between PA and various outcomes and may provide information for specific interventions in older people.

## Background

The demographic change, especially in the industrialized countries, will pose an extreme burden on societies: less caregivers will have to take care of older persons and the state budgets for care will increase [1]. Changing family structures, increased work loads for dual career families and smaller social networks have strongly modified living conditions of older people in these countries [2]. Current robust data are needed, in particular to identify (modifiable) risk factors for the onset of functional decline or disability, to provide new answers to overcome these causes of incident disability but also to identify resources of healthy ageing. In order to address these often multifactorial needs of older persons, population-based cohort studies are essential.

In the last years several cohort studies with older persons have been conducted. Most cohorts have been recruited in Anglo-American countries such as the Women Health and Ageing Study, the Cardiovascular Health Study, the Health ABC-Study or the Canadian Study on Health and Aging [3-6]. However, there are also some studies in Europe such as the Longitudinal Ageing Study Amsterdam [7] or the InChianti Study in Italy [8]. Overall, many modifiable and non-modifiable risk factors for disability have been identified in these projects.

Among intriguing insights into ageing and its consequences, increased physical activity (PA), as a modifiable risk factor, was found to have significant health benefits with regard to multiple endpoints. PA had an effect on cardiovascular health, body composition, metabolism, bone health, psychological well-being, functional capacity and disability [9-16]. However, the assessment of PA in large scale geriatric studies has often been criticized as not appropriate. Most conclusions relied on self-reported instruments with different recall periods [17,18].

Polypharmacy is another potentially modifiable risk factor for diverse health outcomes. The presence of many different drugs (especially centrally acting drugs) but also polypharmacy per se was shown to substantially increase fall risk in older people [19-22] and to significantly increase mortality [23]. Despite these results, concomitant treatment with multiple drugs is based mostly on standard guidelines that have been developed on a single disease basis for mostly younger individuals [24]. Because of these evidence-based guidelines, research projects that include prioritization and medication restriction have been difficult to conduct. However, recent efforts have shown that the use of certain algorithms could lead to a reduction of polypharmacy and an enhanced quality of life [25-27]. In addition, analyses on the association and the effects of multi-medication on certain outcomes were usually complicated by the fact that medication-regimens often change over time. At least in Germany, there is a lack of current information about the incidence of multiple medication (including "Over The Counter" drugs and nutritional supplements).

Falls are one of the most important geriatric syndromes and are closely associated with physical function and PA, which are two of the main objectives of the ActiFE Ulm study. Determinants of falls have been frequently analyzed [28-30]. However, in most studies the fact that fall risk may be modified by the time of exposure has not been adequately examined. In this regard, PA could be an important explanatory variable which may alter established relationships.

To improve our knowledge on associations of the above mentioned and other established risk factors with PA and disability in a cross-sectional and longitudinal sense, we designed the "Activity and Function in the Elderly in Ulm" study (ActiFE Ulm). It is a large population-based single city study, designed as the Geriatric extension of the IMCA - Respiratory Health Survey in the Elderly (IMCA-RHSE) which was implemented in the context of the EU project "Indicators for Monitoring COPD and Asthma - IMCA II". We focused on PA because of the rising importance and modifiability of this risk factor with regard to critical geriatric syndromes such as functional decline, falls and disability. Therefore, PA was assessed using accelerometers, self-report and activity diaries. In addition, in a first continuous follow-up, falls and change of medication/polypharmacy were assessed using a one-year calendar that will be filled out by all participants. Additional research interests of the ActiFE Ulm study group included certain aspects of cognition, inflammation, novel biomarkers, environmental context, pain, nutrition and others. Although the focus of ActiFE Ulm is activity, falls and polypharmacy, the fact that it is conducted together with the IMCA - Respiratory Health Survey in the Elderly will allow studying more relevant associations that go beyond the original settings of both projects.

## Methods/Design

### Project overview

The ActiFE Ulm study is an observational cohort study that was designed as a specific Geriatric extension of the IMCA - Respiratory Health Survey in the Elderly (IMCA-RHSE). Individuals included in the study were asked to complete the IMCA-RHSE Core and Optional questionnaires and measurements to collect baseline data for the ActiFE Ulm longitudinal study. In addition, for the ActiFE Ulm study PA, falls, new methods for medication data collection and some additional geriatric assessments were included. The ethics committee of Ulm University has verified and agreed on the study protocol in January 2009 (Application No. 318/08).

The establishment and definition of a community based study population of older persons will provide a dynamic database that - in addition to the precise study objectives - will allow investigating a wide range of geriatric-related issues, including the relationship between biomarkers, genetic as well as physical, social, emotional and cognitive parameters. This will ultimately increase our insight into the complex relationship of a myriad of promoting factors for successful ageing.

### Aims

#### Physical Activity

**Figure 1:**
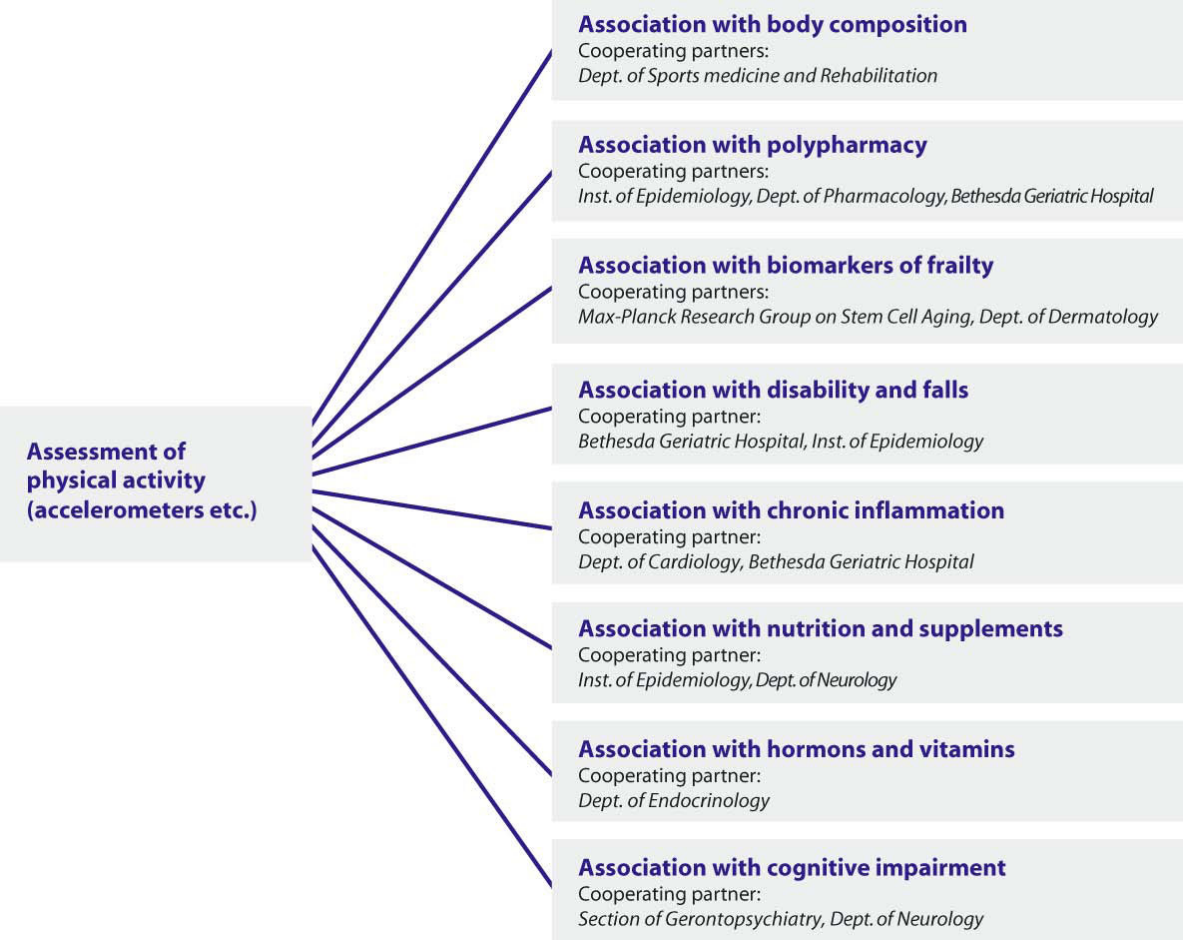
**Planned projects and involved partners using the example of Physical Activity**. Physical activity is in the main focus of the ActiFE Ulm study. Cooperating partners will investigate associations of several distinct risk factors and exposures with physical activity (and other variables as mentioned in the main text).

One aim was to determine reference values for the distribution and extent of PA. Therefore, standardized values for an older population will be established via accelerometer-based PA sensors. In addition, patterns of PA for males and females will be identified and the relationship of PA counts with sedentary behaviour and activity questionnaires will be explored. Selected factors associated with a sedentary life style, such as environmental conditions, weather conditions, cognitive factors, nutritional status, body composition, frailty status, falls history among others will be analyzed (see also figure 1).

The foci of the longitudinal setting are to investigate the impact of PA as well as the influence of change in PA on overall health status and on other health trajectories.

#### (Change of) medication

One major goal of the ActiFE study was to gather information about poly-pharmacy in older adults. In addition, the associations of genetic metabolizing status with disability and PA will be in the focus of this study.

The foci of the longitudinal setting will be the association of medication and change of medication with falls and the effect of interactions within a medication regimen on disability and activity. For that purpose, valid descriptions of all drugs, as well as tracking the change in medication over time, were needed.

#### Falls

The incidence of falls among community dwelling older people will be determined in our study, because, in Germany, there are no validated epidemiologic datasets on falls so far. The effects of certain drug groups, lower extremity physical function and other domains with incident falls will be analyzed. PA as a proxy for the time of exposure (time at risk) will be included as a

The focus of the longitudinal setting will include the impact of falls on physical activity and disability at follow-up.

#### Further aims

The comprehensive assessment protocol and careful phenotypic characterization of the established cohort as well as the cooperation with the IMCA-RHSE study has enabled the creation of a useful database that will facilitate future research activities. This may include the analysis of diverse biomarkers in both blood and urine specimens and their association with prevalent disease, PA levels, functional decline or disability. Using the example of PA, planned analysis and involved partners are demonstrated in figure 1.

### Study design

The IMCA-RHSE has a population-based cross-sectional design. This study is implemented in five EU centres Pisa (Italy), Rome (Italy), Barcelona (Spain), Uppsala (Sweden) and Ulm (Germany). All partners implemented the same set of respiratory questionnaires and measurements. For the centre in Ulm n = 1000 individuals aged between 65 and 90 were randomly selected from a population database. The details of the IMCA-RHSE will be published elsewhere.

The ActiFE Ulm study, which is a single centre longitudinal study, was designed as an add-on to the IMCA-RHSE including a continuous 12-month follow-up measurement of falls and changes in medication.

We intended to detect even small effects (as low as |ρ| = .10) with a still adequate power of 0.8 - maintaining an α-error probability of less than .05%. Taken together these factors resulted in a sample size of at least 780 participants [31]. With regard to expected attrition rates of about 25% per three-year intervals [32] and our intention of at least two follow-up investigations, the sample size was increased by n = 500 to a total of n = 1500 participants.

Some specific geriatric assessments, as suggested by ActiFE Ulm, were also implemented in some centers of the IMCA-RHSE as optional modules (but not including a detailed PA measurement and without the mentioned falls and medication calendar).

**Figure 2:**
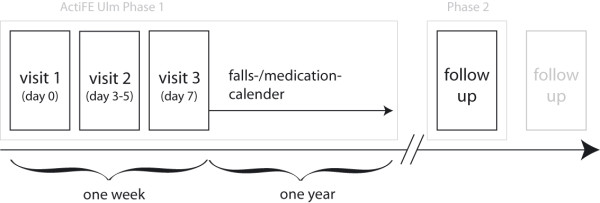
**ActiFE Ulm timeline**. Baseline assessments were conducted within one week per participant. The falls- and medication-calendar constitutes the first longitudinal follow-up (one year follow-up) and belongs to phase one. The projected second phase comprises a 3 year follow-up with a reduced set of assessments but including physical activity.

Concerning ActiFE Ulm, after completed data collection from the one year prospective falls and medication calendar, we will conduct a follow-up (figure 2), giving us the opportunity to estimate causal relationships between exposures and outcomes as described in our objectives.

### Study sample

The target population was located within the greater Ulm, Neu-Ulm and Alb-Donau-Kreis areas, including individuals living in areas with differing urbanization grade. For these regions other types of routinely collected information existed and appropriate information for a random sample selection were available. 1500 participants were recruited from a representative population-based sample (age between 65 and 90).

Recruited were non-institutionalized individuals from the target population (including individuals living in sheltered housing). To obtain the contact addresses, the "Einwohnermeldeämter" (registry offices) of the involved areas were contacted. The sample was stratified for sex and age (three age classes). The aim of over-sampling was to obtain comparable numbers in the respective age groups. This also ensures the presence of older age groups in projected follow-up studies.

The inclusion criteria were: age between 65 and 90 years; informed consent; able to walk through own room (with or without devices). Exclusion criteria were: severe deficits in cognition, vision or hearing that precluded the accomplishment of most assessments. People who had serious German language difficulties were not interviewed.

### Recruitment process

**Figure 3:**
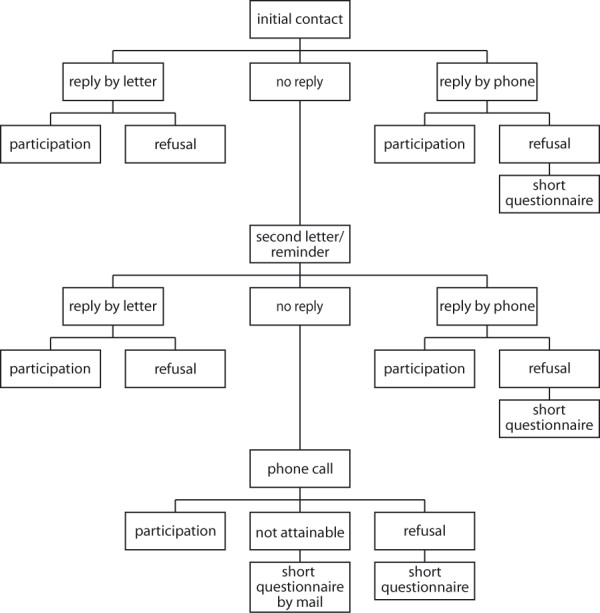
**Recruitment process of the ActiFE Ulm study**. Flowchart on the effort that was made to improve recruitment.

To enhance participation of eligible inhabitants, special effort was dedicated to the recruitment process (see Figure 3). Up to three letters were sent out to each sampled person: The first letter was an invitation to our study including a detailed description of our goals and intentions. Furthermore the legal terms of data and privacy protection were explained. People that were willing to participate were asked to either contact us by phone, e-mail or by the included postage-free return letter. Staff members then contacted participants to make further arrangements for the visits.

If there was no response after the first contact, a second letter was sent out kindly reminding the eligible persons of our study and again asking for participation. As advised by the Ethics Committee, recipients were also notified that they might be contacted by phone if there was public access to their phone number. People that liked to participate after the second letter were asked to contact us by phone or mail and/or were contacted by staff members for further agreements.

If again there was no (negative or positive) response, phone operators were calling those whose telephone numbers were publicly accessible and tried to obtain a definite response about participation or non-participation.

Furthermore, people stating their refusal on the phone were generally asked to answer a small set of questions summarized in a short questionnaire (see table 1). That information will allow us to estimate the possible bias non-respondents may create in our population.

**Table 1:** Complete overview on assessments used in the ActiFE Ulm study

Domain	Type of Assessment	Notes	Origin
Physical activity	Accelerometer (activPAL™)	Continuously over 7 days	From ActiFE Ulm
	Activity diary	Own development	From ActiFE Ulm
	LAPAQ	From the LASA study - Amsterdam	From IMCA-RHSE Optional Q

Physical functioning	Short Physical Performance Battery		From IMCA-RHSE Optional Q
	Basal and Instrumental Activities of Daily Living	From the LASA study including additional items	From ActiFE Ulm
	Handgrip Strength	JAMAR dynamometer	From IMCA-RHSE Optional Q

Falls risk factors	Prospective 1 year falls calendar	Own development	From ActiFE Ulm
	Falls history	3 and 12 months	From ActiFE Ulm
	Level of fatigue, level of dizziness	Numerical rating scales	From ActiFE Ulm
	Falls Efficacy Scale - International; Short Form	Falls-related self efficacy	From ActiFE Ulm

Medication	Medication barcode scan	By hand if no barcode available	From IMCA-RHSE Core Q
	Prospective 1 year "change of medication calendar"	Own development	From ActiFE Ulm

Incontinence	International Consultation on Incontinence Q. - Short Form	Including additional items on overactive bladder and fecal incontinence	From ActiFE Ulm

Affective state	Psychological distress, exhaustion	Numerical rating scales	From ActiFE Ulm
	Hospital Anxiety and Depression Scale		From IMCA-RHSE Optional Q

Medical status and medical history	Medical history/Comorbidity	Functional Comorbidity Index can be derived from items	From IMCA-RHSE Core Q
	Respiratory risk factors including smoking		From IMCA-RHSE Core Q
	Alcohol	Derived from MONICA	From ActiFE Ulm
	Near vision	Snellens' Tumbling E's	From ActiFE Ulm
	Hearing	Interviewers' impression	From ActiFE Ulm
	Height/weight	Digitally measured	From IMCA-RHSE Core M
	Lung function (pre- and post-bronchodilatator spirometry)	Digitally measured	From IMCA_RHSE Core M
	Structured Pain Interview	Abbreviated version	From ActiFE Ulm
	Blood pressure, heart rate and oxygen saturation	Digitally measured	From IMCA-RHSE Core M

Cognition	Mini Mental State Exam		From IMCA-RHSE Optional Q
	Category fluency		From ActiFE Ulm
	Letter sorting		From ActiFE Ulm
	Episodic Memory, word list learning		From ActiFE Ulm
	Episodic Memory, visual Memory		From ActiFE Ulm
	Cognitive estimation tasks		From ActiFE Ulm

Subjective health	Short Form - 12 (Quality of Life)		From IMCA-RHSE Optional Q

Nutrition	Dietary supplements	Vitamins, minerals, yeast, garlic, fibre, others	From IMCA-RHSE Optional Q
	Mini Nutritional Assessment		From IMCA-RHSE Optional Q
	Body Composition	3-point calliper examination	From ActiFE Ulm

Social context	Environmental context	Selected questions from the ENABLE-AGE Project	From IMCA-RHSE Optional Q
	Lubben Social Network Scale	6-item version	From IMCA-RHSE Optional Q
	emotional loneliness	Numerical rating scale	

Health care utilization	Hospital/outpatient health care utilization		From IMCA-RHSE Core Q

Specimens	Blood: EDTA, Serum, Lithium-Heparin	Separate informed consent	From ActiFE Ulm
	Urine	Mid stream morning void	

Short Questionnaire	8 selected questions on COPD/Asthma		From IMCA-RHSE
	2 ADL Questions		From ActiFE Ulm
	1 question on Physical Activity		From ActiFE Ulm
	Demographics		From IMCA-RHSE

ActiFE Ulm = Activity and Function in the Elderly in Ulm

ADL = Activities of Daily Living

COPD = Chronic Obstructive Pulmonary Disease

EDTA = Ethylenediaminetetraacetate

ENABLE-AGE = Enabling Autonomy, Participation and Well-Being in Old Age: The Home Environment as a Determinant for Healthy Ageing

IMCA-RHSE = Indicators for Monitoring COPD & Asthma - Respiratory Health Survey in the Elderly

LAPAQ = Longitudinal Ageing Study Amsterdam Physical Activity Questionnaire

LASA = Longitudinal Ageing Study Amsterdam

MONICA = Monitoring Cardiovascular disease

Q. = Questionnaire

Finally, those people that were neither attainable by phone nor by the first two invitations received a third letter. In this letter, which was clearly marked as the final letter, we included the short questionnaire and asked the person to return it postage free in case of non-participation.

During the progress of the study we realized that the time used for phone recruitment after the second letter was not worth the effort: less than five percent of those contacted could be recruited in addition. This small success was further derogated by the fact that those who agreed to participate were mostly interested anyway, and had just not turned in their consent yet. Response rates showed virtually no difference after we had decided to directly send out a third letter instead of calling.

### Data collection

Participants satisfying the inclusion criteria were contacted by a field worker to make an individual appointment for an interview at home. Participants who did not want to be visited in their home were given the alternative to meet the interviewer in a designated room located at the Bethesda Geriatric Clinic, Ulm.

In total there were three visits incorporated in the ActiFE Ulm study, all to be completed within seven days. The first and last visits were conducted by a study nurse, the visit in between by a physician.

During the first visit the interviewer obtained informed consent from the participant, provided information about the study procedure and conducted the first half of the baseline interview. Furthermore the accelerometer was started and attached to the leg of the participant and the activity diary was handed out. Written information about the physician's visit was distributed. The second visit was conducted by a physician, performing all the clinical assessments that are part of the IMCA-RHSE and just a small set of ActiFE Ulm questions. In the last visit the interviewer accomplished the second half of the baseline interview and detached the activity monitor. Before concluding, the prospective one year falls and medication calendar was handed out and explained extensively. Both study nurse visits lasted approximately 90 minutes each, the doctor's visit was done after an hour. Measures included in the ActiFE Ulm study are described in more detail below and are summarized in table 1. They were thoughtfully selected to achieve the best possible and conclusive set of parameters while also trying not to challenge participants with too time consuming sessions. The importance of this trade-off to maximize clinical research participation and to accomplish valid answers has been pointed out before in a recent article by Marcantonio et al. [33].

### Home interview

The home interview included a selected set of validated questionnaires as well as new instruments that will be evaluated in the course of data analysis. As stated earlier the survey consisted of assessments that were derived from IMCA-RHSE and ActiFE Ulm. The IMCA-RHSE study protocol will be published in detail elsewhere. Briefly, the core questionnaires included questions on socio-demographic characteristics, diagnosis and related respiratory symptoms of asthma and COPD, comorbidity, exposures and potential risk factors related to these two conditions, clinical management (treatment and self management issues), accessibility and use of health services and outcome measures. Clinical assessments that were carried out as part of the IMCA-RHSE study were pulse oximetry, blood pressure, measurement of height and weight and pre- and post bronchodilatator spirometry. The IMCA-RHSE Optional questionnaire and measurements contained assessments that were mainly implemented in the Ulm and Barcelona centres. Further details are described below and listed in table 1. Those assessments derived from IMCA-RHSE are also listed in table 1 in order to give a comprehensive picture of the study.

### The ActiFE Ulm Assessments

#### Physical Activity

To be able to accurately measure physical activity we used accelerometers that record active and sedentary periods during everyday life for one week. Within the seven-day measurement period, activity diaries were kept to collect information about outdoor activities (intended purpose, time spent outdoors, modality, etc.). In order to obtain a comprehensive overview, a PA questionnaire was included and administered during the home visits.

##### PA sensor

PA was predominantly measured using accelerometers with uniaxial movement sensors, called activPAL™ (PAL Technologies Ltd., Glasgow, UK). These monitors were attached to the thigh of the participants to record activity for a continuous period of seven days. Participants were encouraged to contact the study manager if they would experience any problems and they were informed that, in serious cases, the monitor could be removed at any time. The validity of this monitor has been examined numerous times [34,35].

The activPAL™ differentiates activities by the three categories sitting, standing and walking. The definition of sit/lie, stand and move/walk (including the cadence of walking) will provide stable information on the prevalence of activity as well as on detailed aspects such as activity patterns.

##### Activity diary

Outdoor activity was recorded using activity diaries for the duration the monitor was attached. Participants were asked to fill in a questionnaire on a daily basis. That included a question about the persons' well being and the subjectively perceived weather situation as well as a specific query on the times leaving and returning home, the purpose of the activity, the distance covered, the vehicle used and if the participant was accompanied by other persons.

##### The LASA PA Questionnaire

The Longitudinal Aging Study Amsterdam Physical Activity Questionnaire (LAPAQ, Voorrips et al. [36] and Caspersen et al. [37]) examines the frequency and duration of specific types of activity in the past two weeks. The total time spent on PA can be calculated by multiplying the frequency and duration of each activity and summing these values across activities. The LAPAQ has been validated against PA diaries and pedometer counts in a subsample of the LASA population [38] and it has demonstrated good repeatability and moderate validity (similar to other PA questionnaires to date).

### Physical functioning

#### The Short Physical Performance Battery

The Short Physical Performance Battery (SPPB) is used to measure lower extremity function by means of tasks that mimic daily activities. It includes a balance test, a gait speed test that measures the time required to walk four meters at a normal pace and the ability and time to rise from a chair five times. The covered areas represent essential tasks important for independent living and are thus an important outcome or independent variable for all older patients [39]. The SPPB is a frequently used instrument that was shown to be valid not only in identifying and characterizing subjects at the disabled end of the functional spectrum but also in non-disabled, higher functioning older persons [39].

##### (Instrumental) Activities of Daily Living

Another facet of physical functioning is the performance of basic tasks in everyday life (Activities of Daily Living, ADL) and of those that are not necessary for fundamental functioning but ensure independence within the community (concept of Instrumental Activities of Daily Living, IADL).

Emerged from LASA, the ActiFE Ulm study implements the same seven (I)ADL questions as used in LASA. In addition we defined three extra items that cover further aspects and that have proven useful in different settings, resulting in a 10 item IADL scale [40]. The answering format will be comparable to those used in recent functional activity questionnaires [41] and includes the differentiation between dependent and independent.

##### Handgrip strength

The measurement of handgrip strength is an efficient and simple method to estimate overall strength in older people. It has been shown to be highly and independently predictive of functional limitations and disability [42].

### Assessment of falls and of risk factors for falls

The fall history during the past twelve months was asked retrospectively and information on falls was also assessed prospectively by a falls calendar. The ActiFE Ulm study used a falls calendar over a time-period of one year which is filled out by the participants on a weekly basis and sent in to the study center every three months. In order to maximize participants' compliance reminders were sent out every six weeks by the study center. If the calendars did not return within two weeks after they were expected participants were additionally contacted by phone. Thus, recall bias was avoided by the prospective assessment of falls and an impression of previous falls was gained by the assessment of the falls history.

For the assessment of falls-related self-efficacy the Short Form of the Falls Efficacy Scale-International was used. It has been shown to be highly related to previous and subsequent falling [43].

Level of fatigue and level of dizziness may constitute meaningful risk factors for falls [44]. Fatigue was measured using a numerical rating scale from 0 to 10, dizziness using a 5 point Likert scale.

Furthermore, many established risk factors of falls such as muscle strength, balance, cognition, specific comorbidities, loss of vision or the number and type of drugs [29,30] were assessed.

### Medication

#### Barcode Scan

To assess drugs and medications of our population we scanned the barcodes of every pharmaceutical available in the participants' home. In addition, we asked for dosage, frequency and how often in the past twelve months the drug was taken.

In scanning the medication we avoided spelling mistakes, gathered precise information about the pharmaceutical and were able to develop various medication classes for future evaluation. Medications without available barcodes (packages lost, other reasons) were entered by hand.

#### Medication calendar

Analogous to the prospective assessment of falls any change of medication was documented in a second sheet included in the falls calendar. Likewise it was to be filled in on a weekly basis and sent in every three months.

### Hearing and Vision

Impaired hearing has been shown to correlate with poor mobility and may also precede mobility decline [45]. It was assessed using a subjective evaluation by the interviewer using a 4-point Likert scale.

Vision has been found to influence falls in older people; however it is not clear yet if it is an independent risk factor or if it is mediated by PA [46]. In the ActiFE Ulm study near vision is measured using the well known Snellens eye chart (tumbling E's) [47].

### Cognition

#### Category fluency

A test of verbal fluency was applied to measure cognitive flexibility and speed of access to semantic information [48].

#### Episodic memory

Impairment of verbal and visual episodic memory is a hallmark of early dementia, especially dementia of the Alzheimer type. We used word list learning from the CERAD battery [49] in its German adaptation [50]. For assessment of visual episodic memory we composed a new test that required learning a list of abstract symbols.

#### Letter sorting

Working memory and concentration were assessed with the Letter sorting test [51].

#### Cognitive estimation tasks

Cognitive estimation included items with the four dimensions "length", "weight", "quantity" and "time" and required only numerical answers [52,53].

### Psychological distress, exhaustion

Exhaustion and psychological distress were determined on a numerical 10-point rating scale.

General exhaustion over the past four weeks was assessed as well as the specific exhaustion respective to a former completed difficult cognitive task.

### Pain

The "structured pain interview" [54] has been developed for cognitively intact and cognitively impaired geriatric patients suffering from pain. The assessment consists of different questions about location, duration, intensity and frequency of pain. We have implemented a reduced version without the screening questions on cognition and functional capacity; both aspects have been covered by other instruments in our questionnaire.

### Urinary and fecal Incontinence

The "International Consultation on Incontinence Questionnaire - Short Form" (ICIQ-SF) is a brief questionnaire that can be interview-based or self-administered. It was originally developed and validated by Avery et al [55]. It provides a rapid evaluation of the impact of urinary incontinence on quality of life and classifies urinary losses experienced by patients of both sexes [56]. We added a question on urinary urge (overactive bladder) including urge incontinence and two questions on fecal incontinence.

### Nutrition

#### Mini Nutritional Assessment

The Mini Nutritional Assessment (MNA) is a simple and well-validated screening tool for malnutrition in older persons. The 18-item questionnaire includes anthropometric measurements combined with a questionnaire regarding dietary intake, a global assessment of life style as well as the subjective perception of health and nutrition [57].

#### Dietary supplements

Data on the regular use of dietary supplements has been collected. Regular use was defined as 'application for at least for four weeks during the past year'. If the answer was yes, the participant could choose the vitamin or mineral from a list of commonly used vitamins and minerals or they could add other supplements. Participants were asked whether they used preparations with a single component or the combination of different vitamins and/or minerals. Besides vitamins and minerals, information on the supplement use of yeast, garlic and fiber has been collected [58,59].

#### Body Composition/Skin thickness

The abdominal fat percentage was determined by waist circumference, which was measured in a standing position at the level of the umbilicus. Total body fat content was measured using a standardized assessment of three subcutaneous skin-folds according to Jackson and Pollock [60,61] with a Harpenden-Caliper (three different points on one side of the body; for men: chest, abdomen and legs; for women: triceps, abdomen and leg).

#### Alcohol

In order to assess levels of alcohol consumption we implemented the instrument used in the MONICA Augsburg study which is integrated in the WHO's worldwide MONICA project. Each subject is asked how much beer, wine, and spirits he or she drank on the previous workday and over the last weekend. Total alcohol intake is calculated by multiplying weekday consumption by five and adding this figure to weekend consumption. After conversion (1 litre beer = 40 g, 1 litre wine = 100 g, 0.02 litre spirits = 6.2 g alcohol) an average amount of alcohol intake in grams per day can be derived.

This recall method was validated in a subsample of 899 male participants of the first MONICA Augsburg survey in 1984/85, who additionally completed a seven-day dietary record [62].

### Social context

Growing empirical evidence demonstrates the importance of social networks and support to both physical health and well-being among older people [63]. To depict the social networks of our participants, the Lubben Social Network Scale (LSNS) was used. The LSNS is a brief instrument designed to evaluate the size of the social network, perceived support network and perceived confidant network, differentiating between friends and neighbours. It was originally developed in 1988 and was revised in 2002 (LSNS-R) along with an abbreviated version (LSNS-6) which was used in our study [64,65]. A subjective estimation of emotional loneliness was added using a 10 point visual analogue scale.

### Environmental context

The present and past living situation in regard to the level of the floor participants live/lived and the number of steps which have/had to be climbed were asked for in detail. In addition, personal preferences about the individual living situation were assessed using questions from the ENABLE-AGE Project [66].

### Laboratory Measures

If participants had separately agreed, blood was collected during the second visit (physician) in the following order: Serum, EDTA and Lithium-Heparin. Participants who were successfully instructed on visit one, also provided mid-stream urine (morning void).

Baseline laboratory tests included a complete blood cell count, levels of albumin, creatinine, cholesterol, high density lipoprotein, low density lipoprotein, glucose, gamma-glutamyl transferase, uric acid and urea.

Blood and urine specimens were stored for analysis of biomarkers, vitamins, hormones, micronutrients and genetic analyses.

## Discussion

The approach of our project differs considerably from other national and international studies by its predominant focus on activity, medication and falls. With an over-sampling of age groups IMCA-RHSE and ActiFE Ulm is a study with a high number of individuals in the highest age groups. This was needed to adequately picture associations between activity, change of medication, biomarkers of ageing, respiratory disease, falls and their impact on function and autonomy in these steadily growing age-groups.

The cooperation with the IMCA-RHSE will bring mutual benefits combining detailed information on respiratory disease, including high standard pre- and post-bronchodilatator spirometry, and comprehensive geriatric assessments. In the German IMCA-RHSE and ActiFE datasets this will allow detailed analyses on the associations of respiratory diseases with geriatric syndromes and its impact on disability in follow-up studies. Furthermore, certain analyses can be carried out on a larger international dataset, because aspects from the presented ActiFE Ulm study have been included as optional modules in the European IMCA-RHSE. The enriching collaboration with IMCA and the various Ulm University partners led to important choices regarding the selection of assessment instruments used in this study. Our goal was to minimize the impact of the overall data collection on the participants, while attempting to gather all the relevant data that would allow us to adequately examine the objectives of our study. Hence, we had to be concise while not missing important geriatric domains. We believe that this parsimonious approach can be feasible for several parameters like for the assessment of activities of daily living (e.g. practiced in the LASA study) [67] but also for social networking, hearing, vision and other parameters.

The focus on PA implicated a quite detailed look at diverse facets of this important domain. So far, in all large Geriatric studies, PA was assessed via self report which is prone to systematic error and typically associated with poor validity [17,18,68,69]. In a study on physical frailty in hospitalized older people, engagement in PA was even estimated using proxy items that reflect rather functional and physical capacities than real PA [70,71]. Moreover, PA was rarely available as a continuous variable, considerably reducing statistical power. Recent data suggest that adequate assessment of PA in older people has to involve objective measurements [18]. However, the direct measurement of PA is challenging, often costly and, for large studies, not all standardized PA assessments are feasible. For example, the standard double labeled water technique and the measurement of oxygen consumption by minute to minute heart rate or ventilation masks can only be used in laboratory settings and are impractical for the assessment during everyday live in the community [72,73]. It is now widely accepted that the most attractive and objective method to date are accelerometer- or pedometer-based sensors [18].

To be able to study patterns of PA in 1500 participants in conjunction with overall activity counts we had to find a monitor that provided enough detail with a reasonable amount of storable data. We decided to use the activPAL™ monitor (PAL technologies Ltd., Glasgow, Scotland) for several reasons: First of all this monitor is small and not compromising quality of life for the recording time span. Second, since it is attached to the leg using adhesive film unlike other monitors that are integrated in a belt it is certain to remain on the leg for the intended period of time. Third, it is a uniaxial sensor with a reasonable amount of data recorded over a seven days period (without the need to change batteries). Fourth, being attached to the thigh, it allows for the differentiation of sedentary (sitting, lying) and active (standing, walking) periods.

When developing the activity diary we decided to focus on outdoor activities because this reflected the most important contribution to overall activity and it is better remembered as compared to indoor activity (which involves high assessment efforts and misclassification). In addition, we particularly developed the activity diary to complement the objective measurement of PA and to add specific details on patterns of activity that can contribute to an exhaustive analysis of PA. Since the relationship of PA with health outcomes may differ by the type of activities [74,75], we also needed to discriminate between different intensities of activity. A simple method to take intensity into account is to distinguish sports activities from non-sports activities. For that purpose the LASA Physical Activity Questionnaire (LAPAQ) was incorporated into the study protocol.

PA is often understood as some kind of (even low intensity) sports. However, studies showing that even a one hour walk per week already results in a reduced risk for cardiovascular events should not be ignored [76]. Therefore, habitual PA-levels could be of interest to both researchers and health care authorities allowing disentangling the patterns of PA, different for age, sex and/or social status. This could be helpful in terms of future preventive measures and programs. In order to monitor future interventions, standardized normative values across different age-groups should be of major interest. This involves a comparison of the accuracy of current activity questionnaires with a standardized activity sensor. Moreover, prevailing hypotheses of the above mentioned associations of PA with other health outcomes or with healthy ageing could be verified using these new techniques.

At present, some of the results on PA are conflicting. For example the association of falls and disability in the context of PA is largely unknown [77] although the incidence of falls among community-dwelling older people aged 65 years or older is approximately 30% per year [28] and the individual (fractures/immobility) [29] and economical costs are substantial: therapy-costs of the proximal hip fractures in Germany run up to € 500 million per year [78]. Therefore, there is a need for strategies to prevent falls and fall-related injuries in older people on the individual and the community level. This is, however, problematic since both high and low levels of PA have been linked to an increase in falls risk (in a linear or U-shaped relation) and the association could be highly influenced by physical function. To our knowledge only few studies have been designed in a (methodologically) adequate way to make any assumptions about this relation [77,79,80].

Associations between drug groups and falls have been previously reported [20-22]. Most data derived from cross-sectional or retrospective studies. In the ActiFE Ulm study change of medication and incidence of falls were assessed both simultaneously and prospectively. To the best of our knowledge this has not been done before. In addition, most research focused on antecedents of falls whilst there is less information on the long-term consequences of falls other than injury or mortality.

With regard to polymedication it is well known by both clinicians and researchers alike that potentially appropriate medications for one diagnosis could worsen other diagnoses. Because of the difficulty to maximize benefits for all conditions in comorbid older persons it is essential to carefully weigh risks and benefits. E.g. fall risk, hypertension and adverse medication effects represent frequent trade-off situations in older adults [81]. Especially associations of PA and polypharmacy or centrally acting medications have not been subject to intense research activities [82]. Polypharmacy per se has been associated with falls and even mortality in older persons [22,23,83].

When looking at medication effects, potential interactions (CYP-enzymes, the individuals' metabolic status such as rapid versus slow metabolizers, etc.) have to be considered. These complex associations demand large cohort sizes and precise outcome assessments. To our knowledge, a comparable prospective assessment of medication change over one year has not been performed before in an older population. With a detailed assessment of medication use we will be able to evaluate the influence of inappropriate medication and the influence of medication change on all mentioned outcome measures.

The ActiFE Ulm study includes both performance-based and self-rated assessments to measure physical function as suggested in a recent consensus-paper [84]. Questionnaires regarding physical performance add an important aspect to the overall description of physical functioning: while outcomes of performance tests clearly depend on one concrete situation, questionnaires are able to depict physical functioning as a subjective perception averaged over a longer period of time.

The study started as a cross-sectional project in cooperation with the IMCA-RHSE study extended by a continuous one year follow-up which was restricted to falls and change of medication. However, an established cohort with such a high number of older and very old individuals offers unique opportunities for longitudinal investigations. Therefore, a three years follow-up with focus on PA, disability, mortality and institutionalization is projected as outlined in figure 2. Further follow-up studies and auxiliary studies are desirable, already in preparation or will be planned in the near future.

We hope that the ActiFE Ulm study will shed light on the accuracy of reported PA, will allow for the estimation of standard PA counts in older persons, their distribution over time and on the impact of PA on falls or its association with certain medication changes. This again could influence public health PA programs and help to find the right treatment for these often poly-morbid older persons. The detailed fall-calendars will help analyzing these rather intimate personal stories. The ActiFE Ulm Team is looking forward to the end of the first recruitment period and invites interested Geriatric or Epidemiologic researchers to participate in our study.

## Competing interests

The authors declare that they have no competing interests.

## Authors' contributions

MDD designed and organized the ActiFE Ulm study and drafted the manuscript. SF organized the ActiFE Ulm study and drafted the manuscript. KR designed and organized the ActiFE Ulm study and helped to draft the manuscript. GW designed the ActiFE Ulm study and critically revised the manuscript. TN is principal investigator, conceived of the ActiFE Ulm study and critically revised the manuscript. EDT designed the IMCA-RHSE study and critically revised the manuscript. RP is principal investigator of the IMCA-RHSE (Ulm) and the ActiFE Ulm studies, conceived of the ActiFE Ulm study, and critically revised the manuscript. The ActiFE Ulm study group helped to organize the study, contributed to specific assessments and approved the manuscript. All authors read and approved the final manuscript.

## Pre-publication history

The pre-publication history for this paper can be accessed here:

http://www.biomedcentral.com/1471-2318/10/50/prepub
